# Elevated serum CA19-9 level is a promising predictor for poor prognosis in patients with resectable pancreatic ductal adenocarcinoma: a pilot study

**DOI:** 10.1186/1477-7819-12-171

**Published:** 2014-06-02

**Authors:** Qian Dong, Xiang-hong Yang, Yao Zhang, Wei Jing, Li-qiang Zheng, Yun-peng Liu, Xiu-juan Qu

**Affiliations:** 1 Department of Oncology, Shengjing Hospital of China Medical University, Shenyang, P.R. China; 2 Department of Pathology, Shengjing Hospital of China Medical University, Shenyang, P.R. China; 3 Department of Ultrasound, Shengjing Hospital of China Medical University, Shenyang, P.R. China; 4 Library, Shengjing Hospital of China Medical University, 36 Sanhao Street Heping District, Shenyang 110004, P.R. China; 5 Department of Oncology, the First Affiliated Hospital of China Medical University, 155 Nanjing North Street, Heping District, Shenyang P.R. 110001, China; 6 The Shengjing Hospital, China Medical University, 36 Sanhao Street Heping District, Shenyang 110004, P.R. China

**Keywords:** Pancreatic ductal adenocarcinoma (PDAC), Carbohydrate antigen 19-9 (CA19-9), Prognosis, Overall survival (OS)

## Abstract

**Background:**

Pancreatic ductal adenocarcinoma (PDAC) is one of the most aggressive human cancers. Several studies have reported that the carbohydrate antigen 19-9 (CA19-9) level is a useful marker for predicting the prognosis for PDAC after resection. However, the cutoff value of CA19-9 used to predict prognosis varied among these reports. The aims of this study were to evaluate whether the serum CA19-9 level is a significant predictor for survival and to determine the optimal cutoff value of CA19-9 for predicting prognosis.

**Methods:**

A total of 120 consecutive patients who underwent surgery for potentially resectable primary PDAC were retrospectively analyzed. The variables included the following: age, sex, the location of the tumor, the maximal tumor size, the histological differentiation, the margin status, the tumor stage, serum CA19-9 levels, and serum total bilirubin (TBil) levels.

**Results:**

The overall 1-year survival rate was 62.5%. The receiver operating characteristic (ROC) curve indicated a significant result for the level of CA19-9 in predicting death within 1 year after surgery (Area under the curve (AUC), 0.612; 95% confidence interval (CI), 0.505-0.720; *P* = 0.040). The optimal cutoff point was 338.45 U/mL (sensitivity, 60.0%; specificity, 66.7%; accuracy, 64.2%). The strongest univariate predictor among the categorized CA19-9 values was CA19-9 greater than or equal to 338.45 U/mL. In the multivariate Cox proportional hazards mode analysis, the serum CA19-9 level, age and the histological differentiation were significant independent prognostic factors that were associated with the overall survival.

**Conclusions:**

The preoperative elevated CA19-9 level is a promising independent factor for predicting a poor prognosis in PDAC, and the optimal cutoff value is 338.45 U/mL.

## Background

Pancreatic cancer is one of the most aggressive human cancers and is characterized by a rapid tumor spread and a dismal prognosis [1]. By the time of diagnosis, fewer than 15% of patients can be offered a potential curative treatment, and up to 30% of the patients die within 12 months [2,3]. Additionally, the 5-year survival rate of patients with potentially resectable pancreatic cancer was reported not to exceed 20% [4]. It is extremely important to precisely predict the prognosis after tumor resection for the assessment of the therapeutic effect, for the consideration of administering adjuvant therapy, and to provide information to patients.

To date, several studies have reported that the preoperative carbohydrate antigen 19-9 (CA19-9) level, which is a tumor-associated antigen that requires the expression of a sialylated Lewis blood group antigen for expression, is a useful marker for predicting prognosis after resection [5-13]. However, the cutoff value of CA19-9 used to predict prognosis varied among these reports. A value of 37 U/mL has been frequently used as the cutoff value in previous studies [7,10,14,15]. The normal physiological level of serum CA19-9 is defined as under 37 U/mL, which was estimated based on the standard deviation of the distribution of serum CA19-9 levels among normal persons. Serum CA19-9 levels are elevated in 70% to 80% of pancreatic cancer patients [16] and may be more suitable for the diagnosis of pancreatic cancer rather than for predicting the prognosis for the patients with established diagnosis. Thus, it is still uncommon for clinicians to predict prognosis with preoperative CA19-9 values.

The aims of this study were to evaluate whether the preoperative CA19-9 value is a significant predictor for survival and to determine the optimal cutoff value of CA19-9 for predicting the prognosis in PDAC.

## Methods

### Patients and methods

From 1 January 2009 to 28 February 2011, 139 consecutive patients underwent surgery for potentially resectable primary pancreatic ductal adenocarcinoma (PDAC) at the Shengjing Hospital of China Medical University. In all of the patients, the PDAC was histologically confirmed. Patients with other pancreatic malignancies, such as intraductal papillary mucinous adenocarcinoma, acinar cell carcinoma, adenosquamous carcinoma, mucinous carcinoma, and malignant endocrine tumors, were excluded from this study. The patients had not received chemotherapy or radiation therapy prior to surgery.

The variables included the following: age, sex, the location of the tumor (head, body or tail of the pancreas), the maximal tumor size (cm), histological differentiation (well, moderately or poorly differentiated), the margin status (positive or negative), the tumor stage, the node stage, the TNM stage, preoperative serum CA19-9 levels, and preoperative serum total bilirubin (TBil) levels. The maximal tumor size was defined as the maximum diameter on pathological analysis. The margins assessed included the pancreatic resection margin, the biliary margin, the posterior margin, the retroperitoneal margin, and the mesenteric margin. The tumors were staged according to the TNM criteria of the American Joint Committee on Cancer (AJCC) 2010 staging system [17].

All of the serum CA19-9 levels were measured within 2 weeks before surgery with an electrochemiluminescence immunoassay using the Roche Cobas E601 (Roche, Switzerland) immunoassay system. The upper limit of the normal reference value for CA19-9 is 37 U/mL. The preoperative TBil levels were detected concurrently with the CA19-9 levels using a vanadic acid oxidation method with the Abbott ARCHITECT ci1600 (Abbott, USA) automatic analyzer. Altered biliary excretion, for which bilirubin is reasonable marker, has been documented to occur at levels 1.5× the upper limit of the normal reference value or at a level of approximately 34.2 μmol/L (2.0 mg/dL) [18].

The patients themselves or the clinicians and relatives of the patients were contacted by telephone and interviewed for patient survival or the documented day of death. The final survival data were collected on May 15, 2013. The median follow-up period was 15.0 months (range, 1.4 to 52.0 months). Adjuvant chemotherapy with a standard regimen was performed on all of the patients who were able to tolerate postoperative chemotherapy regardless of the margin status or tumor stage.

Nineteen of the 139 patients were excluded from the present analysis for the following reasons: six patients (4.3%) died in the hospital within 30 days after the surgery; the clinicopathological characteristics and prognosis were not available for six patients (4.3%); and seven patients (5.0%) had a preoperative CA19-9 value of <2 U/mL and were judged to be non-secretors of CA19-9 [6]. Ultimately, 120 patients were analyzed. All of the patients’ data were retrospectively reviewed after approval from the Research Ethics Committee of Shengjing Hospital of China Medical University.

### Statistical analysis

The continuous variables such as age, serum CA19-9 and TBil levels, and follow-up periods were expressed as medians with ranges. The patients who were alive at the last follow-up were censored. The comparisons between clinicopathological characteristics and the CA19-9 values were performed with a Mann-Whitney *U* test or a Kruskal-Wallis *H* test if the grouping variables were more than two. Overall survival (OS) was defined as the time from the date of the surgery to either the date of death from any cause or the last contact, that is, the date of the last follow-up visit. The cutoff points to categorize the serum CA19-9 levels were obtained as follows: (1) the upper limit of the normal reference value for CA19-9 (37 U/mL); (2) the mean value of CA19-9; (3) the median value of CA19-9; (4) the receiver operating characteristic (ROC) curve was used to assess the ability of CA19-9 to predict for 1-year death and to determine the optimal cut-off point of CA19-9; (5) additional cutoff points were chosen based on data previously published by other groups (200 U/mL and 1000 U/mL) [7,19,20]. The categorization divided the patients into those less than *versus* those greater than or equal to the indicated cutoff points. Different categorizations of the preoperative values of CA19-9 divided by the above six cutoff points were examined. The survival curve was calculated using the Kaplan-Meier estimate. The survival differences between subgroups of patients were analyzed by the log-rank test. The strongest univariate predictor among the categorized serum CA19-9 measurements was chosen. The multivariate Cox proportional hazards model (forward) was fitted using all of the clinical and pathological variables, which included age, gender, the location of the tumor, the maximal tumor size, the histological differentiation, the surgical margins, the pT category, the pN category, the pTNM category, serum TBil level, and CA19-9 with the optimal cutoff value. The corresponding hazard ratios (HRs) and their 95% confidence intervals (CIs) were calculated. SPSS software version 13.0 (SPSS Inc., Chicago, IL, USA) was used for the statistical analysis. Two-sided *P* values less than 0.05 were considered to be statistically significant.

## Results

### Demographic and clinicopathological characteristics

One hundred and twenty patients were included in this study. Table 1 lists the demographic and clinicopathological characteristics of these 120 patients. The median age was 60 years (range, 35 to 80 years), and 67 (55.8%) of the patients were men. The tumors of 92 (76.7%) patients were primarily located at the pancreas head, and 28 (23.3%) tumors were located at the pancreas body or tail. The median size of the tumors was 4.0 cm (range, 1.2 to 10 cm). Regarding the histological differentiation, 41 (34.2%) patients had a well-differentiated tumor, 68 (56.7%) patients had a moderately differentiated tumor, and 11 (9.1%) had a poorly differentiated tumor. One hundred and fourteen (95.0%) patients had negative surgical margins, and 39 (32.5%) patients were positive for nodal metastasis. Sixty-two (51.7%) patients had tumors that extended beyond the pancreas.

**Table 1:** The relationship between serum CA19-9 levels and clinicopathological factors in 120 cases of PDAC treated by surgical resection

**Characteristics**	**Number (*n*** **= 120)**	**CA19-9 (Median, U/mL)**	***P*** **value**
Age (years): Median (Range)	60 (35-80)		
<60	57 (47.5%)	297.20	0.879
≥60	63 (52.5%)	243.00	
Gender			
Male	67 (55.8%)	280.51	0.417
Female	53 (44.2%)	294.70	
Location of tumor			
Head	92 (76.7%)	294.95	0.782
Body or tail	28 (23.3%)	242.30	
Maximal tumor size (cm): Median (Range)	4.0 (1.2-10)		
<4.0	48 (40.0%)	227.65	0.193
≥4.0	72 (60.0%)	301.05	
Differentiation			
well	41 (34.2%)	130.40	0.024
moderately	68 (56.7%)	467.00	
poorly	11 (9.1%)	208.77	
Surgical margins			
Negative	114 (95.0%)	295.95	0.087
Positive	6 (5.0%)	109.23	
pT category			
pT1 + pT2	58 (48.3%)	205.10	0.055
pT3 + pT4	62 (51.7%)	328.60	
pN category			
pN0	81 (67.5%)	250.00	0.403
pN1	39 (32.5%)	328.80	
pTNM category			
I	44 (36.7%)	205.10	0.161
II	53 (44.2%)	304.90	
III	23 (19.1%)	558.80	
Serum total bilirubin (μmol/L): Median (Range)	29.80 (2.80-507.30)		
<34.2 μmol/L (2.0 mg/dL)	63 (52.5%)	187.90	0.025
≥34.2 μmol/L (2.0 mg/dL)	57 (47.5%)	332.80	

### The clinicopathological characteristics *versus* serum CA19-9 levels

As shown in Table 1, the serum CA19-9 levels were strongly associated with the histological differentiation, and the median CA19-9 levels increased with the tumor grade (*P* = 0.024). The median CA19-9 value was higher in the group with a TBil level of ≥34.2 μmol/L than in the group with a TBil level <34.2 μmol/L (*P* = 0.025). The median CA19-9 levels were different between the patients with T1/T2 and T3/T4, but no statistical significance was observed (*P* = 0.055).

### Comparisons among the cutoff points of CA19-9 values

**Figure 1:**
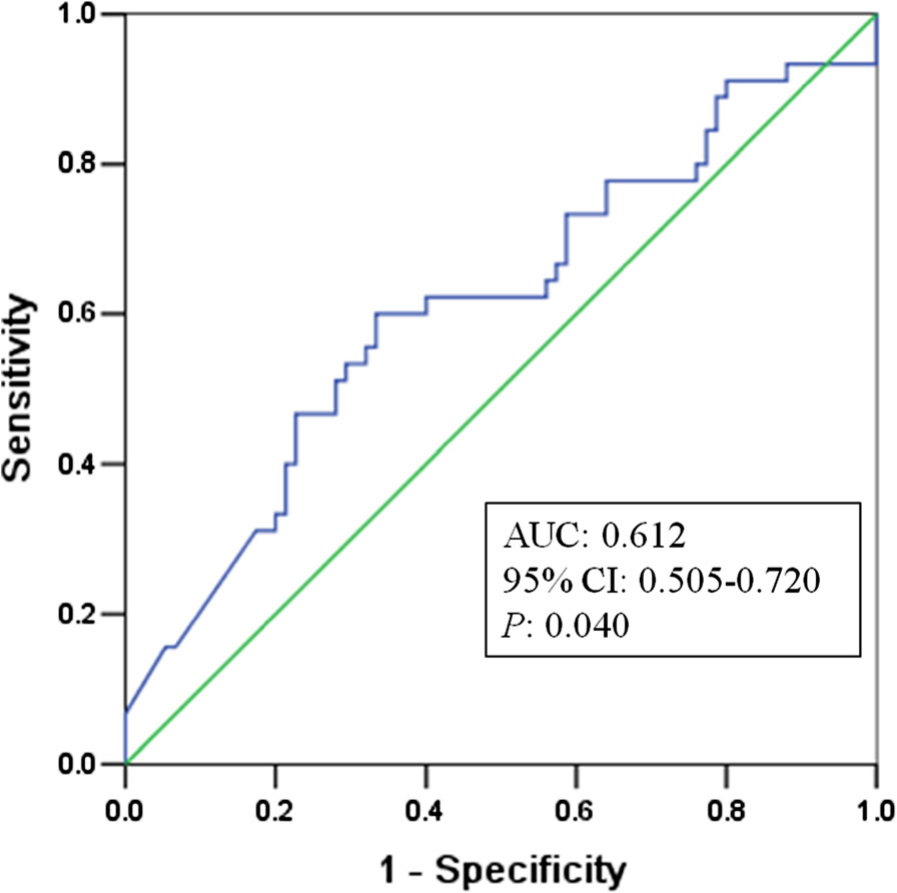
**ROC curve for the 1-year death.** AUC with the 95% CI is noted in the graph inset; the *P* value is with regard to testing the prognostic accuracy against the null hypothesis (area = 0.5).

**Figure 2:**
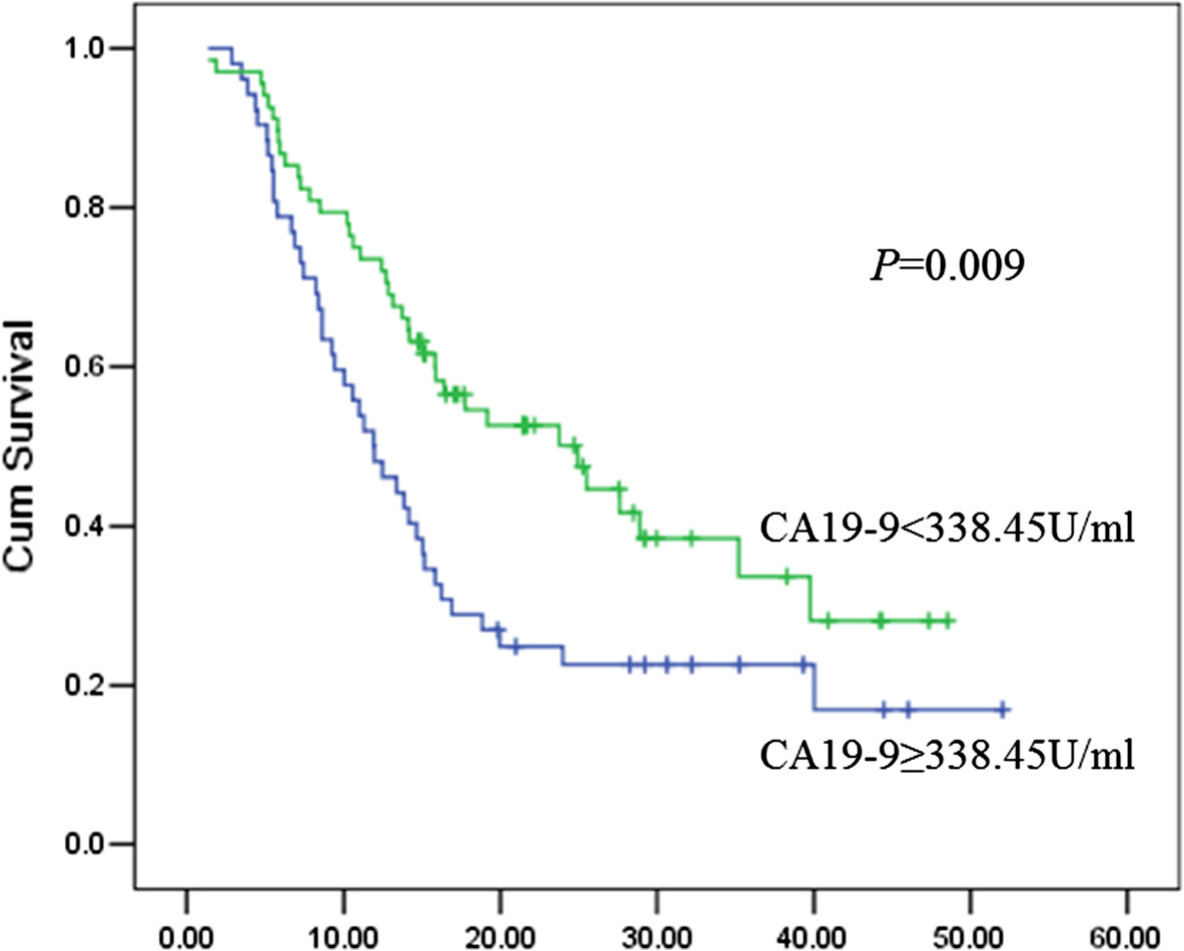
**Comparison of the overall survival between the two groups separated by the CA19-9 cutoff value of 338.45 U/mL.** A significant difference in survival was revealed by the log-rank test (*P* = 0.009).

The mean and the median values of CA19-9 were 617.31 U/mL and 287.61 U/mL, respectively. The overall 1-year survival rate was 62.5%. Forty-five patients died within the first year after the surgery to resect the tumor. Figure 1 shows the ROC curve for the 1-year death and the CA19-9 levels. The ROC analysis indicated a significant result for the level of CA19-9 in predicting death within 1 year after surgery (Area under the curve (AUC), 0.612; 95% CI, 0.505-0.720; *P* = 0.040). The optimal cutoff point was 338.45 U/mL (Youden index, 0.27; sensitivity, 60.0%; specificity, 66.7%; accuracy, 64.2%). The sensitivity, specificity, and accuracy for 37 U/mL as the cutoff point were 91.1%, 18.7%, and 45.8%, respectively. Table 2 summarizes the univariate tests for all of the categories considered for overall survival. As shown in Table 2, the strongest univariate predictor among the categorized CA19-9 values was a level of CA19-9 <338.45 U/mL. The patients with a level of CA19-9 <338.45 U/mL had a median overall survival time of 24.9 months compared with 11.9 months for patients with a level of CA19-9 ≥338.45 U/mL (log rank *x*^*2*^, 6.868; *P* = 0.009) (Figure 2).

**Table 2:** Univariate analysis of the overall survival of two groups divided on the basis of the CA19-9 cutoff value in patients with PDAC treated by resection

**Cutoff value (U/mL)**	**Number (*n*** **= 120)**	**Median OS (months)**	**Log rank** ***x***^***2***^	***P*** **value**
<37	18	21.6	6.647	0.010
≥37	102	14.2		
<200	52	19.2	2.105	0.147
≥200	68	13.9		
<287.61	60	19.2	3.923	0.048
≥287.61	60	12.7		
<338.45	68	24.9	6.868	0.009
≥338.45	52	11.9		
<617.31	79	19.2	4.696	0.030
≥617.31	41	12.0		
<1,000	93	15.9	0.827	0.363
≥1,000	27	12.0		

### Multivariate Cox proportional hazards model analysis of the prognostic factors for overall survival

The multivariate Cox proportional hazards model (forward) was fitted using all of the clinical and pathological variables, which included age, gender, the location of the tumor, the maximal tumor size, the histological differentiation, the surgical margins, the pT category, the pN category, the pTNM category, serum TBil level, and serum CA19-9 level. In the multivariate Cox proportional hazards model (forward) analysis, a CA19-9 level ≥338.45 U/mL (HR, 1.961; 95% CI, 1.241-3.099; *P* = 0.004), an age ≥60 years (HR, 0.554; 95% CI, 0.351-0.874; *P* = 0.011), a histologically moderately differentiated tumor (compared with well-differentiated, HR, 2.108; 95% CI, 1.251-3.551; *P* = 0.005), and a histologically poorly differentiated tumor (compared with well-differentiated, HR, 4.393; 95% CI, 1.932-9.986; *P* = 0.000), were significant independent prognostic factors associated with overall survival (Table 3).

**Table 3:** **Significant independent prognostic factors for overall survival in patients with PDAC treated by resection on multivariate analysis**^**a**^

**Characteristics**	**Category**	**Hazard ratio**	**95% CI**	***P*** **value**
Age (years)	≥60 *vs.* <60	0.554	0.351-0.874	0.011
Histological differentiation	well	1		0.001
	moderately	2.108	1.251-3.551	0.005
	poorly	4.393	1.932-9.986	0.000
CA19-9 (U/mL)	≥338.45 *vs.* <338.45	1.961	1.241-3.099	0.004

^a^The multivariate Cox proportional hazards model (forward) was fitted using all of the clinical and pathological variables, which included age, gender, location of tumor, maximal tumor size, histological differentiation, surgical margins, pT category, pN category, pTNM category, serum TBil level, and CA19-9 level.

## Discussion

The tumor-associated CA19-9 antigen has been accepted as the most meaningful tumor marker for pancreatic cancer. Its clinical usefulness in the diagnosis [21], the assessment of resectability [14], monitoring the progression [11] and determining the prognosis of pancreatic cancer has been reported [7-13]. Ferrone *et al.*[20] reported that the preoperative level of CA19-9 was not an independent prognostic factor in patients with resectable pancreatic adenocarcinoma. However, a contradictory result has been revealed by the Waraya [10], Hartwig [22], and Hirakawa [15] groups, arguing that the preoperative CA19-9 level is an independent prognostic factor on multivariate analysis. The result from our study is consistent with the latter findings. According to the ROC curve analysis and the multivariate Cox proportional hazards model analysis, we found that the preoperative CA19-9 level is a promising predictor for the overall survival or prognosis of PDAC.

Although it has been accepted that the level of CA19-9 can be used as a valuable predictor for the prognosis of PDAC, the cutoff value of CA19-9 is still controversial. Waraya *et al.*[10] and Hirakawa *et al.*[15] reported that a level of CA19-9 >37 was the independent prognostic factor. Berger *et al.*[7] reported that undetectable preoperative CA19-9 levels and a value of CA19-9 >200 U/mL were related to survival. A value of 50 U/mL was used by Kang *et al.*[23], while Hartwig *et al.*[22] reported that a level of CA19-9 ≥400 was an independent negative predictor. The cutoff values of CA19-9 used in previous studies were varied. Among them, a value of 37 U/mL was most frequently used. This cutoff value is also recommended as the upper limit of the normal range for CA19-9, which is elevated in 70% to 80% of pancreatic cancer patients. However, in our clinical practice, it has been found that a few patients did not yet have poor prognoses as predicted after a regular surgical resection and routine chemotherapy, although a level of CA19-9 >37 U/mL was observed in these patients. Using 37 U/mL of CA19-9 as the cutoff value may be more suitable for the diagnosis of pancreatic cancer rather than predicting a prognosis for the patients with an established diagnosis. In this study, an attempt to determine the optimal cutoff value was carried out, with comparisons made among six cutoff points. The optimal cutoff point was 338.45 U/mL based on the ROC curve analysis, and the specificity and accuracy were increased compared with using 37 U/mL as the cutoff point. Moreover, the relationship between these six CA19-9 cutoff points and overall survival was assessed with univariate analysis. The results indicate that the strongest univariate predictor is a level of CA19-9 ≥338.45 U/mL. According to the multivariate Cox proportional hazards model analysis, a preoperative CA19-9 value ≥338.45 U/mL is also a significant independent prognostic factor for a poor prognosis in PDAC.

As an important marker of PDAC, the serum CA19-9 level can be influenced by other factors. One of the most common factors may be the histological differentiation of the tumor cells, which is considered to be a characteristic of malignancy. It has been suggested that an altered secretion or distribution pattern in pancreatic tumors leads to the differential levels of the tumor-associated CA19-9 antigen in the serum [24]. Thus, the CA19-9 levels may vary with the characteristics of the tumor cells. In this study, the CA19-9 levels were strongly associated with histological differentiation. Similar to the study by Rahbari *et al.*[25], the age, differentiation, and the CA19-9 levels were revealed to be independent predictors for overall survival with a multivariate analysis. The CA19-9 level has been reported to correlate with tumor burden [18], but in our present study, a contradictory result was observed: the CA19-9 levels did not correlate with the tumor size or the amount of tumor cells. The findings indicate that CA19-9 is a characteristic of the tumor cells in PDAC, which may reflect the secretion capacity and the malignant biological behavior of the tumor.

Although, the median CA19-9 value was higher in the group with a TBil level ≥34.2 μmol/L than in the group with a TBil level <34.2 μmol/L, the TBil level was not a prognostic predictor of overall survival according to the multivariate analysis. In addition, for the median serum TBil level, there were no differences between the patients with a CA19-9 level ≥338.45 U/mL and those with a level of CA19-9 <338.45 U/mL (*P* = 0.062). Similar to our results, Hartwig *et al.*[14] reported that hyperbilirubinemia did not markedly affect the CA19-9 levels.

In the present study, neither the tumor size nor grade was the independent prognostic predictor, which was consistent with Berger *et al.*[26] but discrepant with Waraya *et al.*[10]. The relationships between the tumor size or grade and the outcome of PDAC have produced mixed results. Though the tumor size and grade may represent some aspects of the malignancy of the tumor, there are also other aspects that determine the malignancy of the tumor, for example, the histological differentiation. In this study, the most significant prognostic predictor was the histologically poorly-differentiated tumor (HR, 4.393; *P* = 0.000). Thus, the relationships between the tumor size or grade and the outcome of PDAC could be clarified in the further prospective validation, including an adequate number of patients.

Ferrone *et al.*[20] reported that the surgical margin was not an independent prognostic predictor for the patients with resectable PDAC, which was consistent with the result of the present study. However, Kinsella *et al.*[27] reported that the surgical margin was an independent prognostic factor. The discrepancies among the studies that assess the relationship of surgical margin and survival may be due to the different therapeutic approaches and pathology reporting. The contents of the margin assessed in the study of Kinsella *et al.* contained pancreatic neck, uncinate process, the common bile duct, portal vein groove, anterior soft tissue and posterior soft tissue margins of excision, which were different from that of the present study. On the other hand, the extent of the margin clearance was associated with the survival [28]. In addition, the molecular-genetic evaluation of surgical margin could improve pathological staging and prognostic evaluation of patients with PDAC [29]. Kim *et al.*[29] reported that K-ras gene mutation in histologically negative surgical margins associated with poor outcome of patients with PDAC. Therefore, the relationships between the surgical margin and the prognosis of patients with resectable PDAC could be investigated in the further prospective studies, in which all of the factors above mentioned should be taken into account.

Lastly, inevitable limitations exist in the present study as a pilot study. First, the follow-up period is one of the greatest concerns. PDAC is the type of disease that has a dismal prognosis with a 5-year survival rate of approximately 5% [30]. Prospective validation, including an adequate number of patients, is required to clarify the relationship between the preoperative CA19-9 value and prognosis. Another concern of this study is the CA19-9 factor; the level of CA19-9 may oversimplify the prediction of prognosis for PDAC, and in the future, more extensive factors should be explored.

## Conclusions

In conclusion, a preoperative CA19-9 value ≥338.45 U/mL was shown to be a promising independent factor for a poor prognosis in PDAC on univariate and multivariate analysis. The preoperative CA19-9 level may be clinically useful for a prognostic valuation in cases of resectable PDAC. CA19-9 levels appear to have a useful place in the strategic planning for the management of patients with PDAC.

### Consent

Written informed consent was obtained from the patient for the publication of this report and any accompanying images.

## Abbreviations

AJCC: American Joint Committee on Cancer; AUC: Area under the curve; CA19-9: Carbohydrate antigen 19-9; CI: Confidence interval; OS: Overall survival; PDAC: Pancreatic ductal adenocarcinoma; ROC: Receiver operating characteristics; TBil: Total bilirubin.

## Competing interests

The authors declare that they have no competing interests.

## Authors’ contributions

QD carried out the records collection and analysis, and drafted the manuscript. X-hY participated in the design of the study. YZ and WJ participated in records collection. L-qZ participated in the statistical analysis. Y-pL and X-jQ participated in its design and helped to draft the manuscript. All authors read and approved the final manuscript.

## Authors’ information

All the work was done in the Shengjing Hospital of China Medical University, Shenyang, China P.R.; and the paper was not presented in any meeting.
